# Lesion measurement on a combined “all-in-one” window for chest CT: effect on intra- and interobserver variability

**DOI:** 10.1186/s40644-019-0262-0

**Published:** 2019-11-29

**Authors:** Annemiek Snoeckx, Jeroen Cant, Caro Franck, Elisa Luyckx, Ken Carpentier, Simon Nicolay, Astrid Van Hoyweghen, Maarten J. Spinhoven, Pieter Vuylsteke, Paul M. Parizel

**Affiliations:** 1 Department of Radiology, Antwerp University Hospital and University of Antwerp, Wilrijkstraat 10, 2650 Edegem, Belgium; 2 Agfa Medical Imaging, Septestraat 27, 2640 Mortsel, Belgium

**Keywords:** Diagnosis, Thoracic neoplasms, Computed tomography, Window, Postprocessing

## Abstract

**Purpose:**

A newly developed image processing technique fuses conventional windows into a single ‘All-In-One’ (AIO) window. This study aims to evaluate variability of CT measurement of lesions in thoracic oncology patients on this novel AIO-window.

**Methods:**

Six radiologists with different levels of expertise measured 368 lesions of various size, origin and sharpness. All lesions were measured twice on the AIO-window and twice on the conventional window settings. Intraclass correlation coefficients and Bland-Altman plots were used to assess intra- and interobserver variability.

**Results:**

Overall intra-observer agreement for lesion diameters on the AIO-window and conventional window settings was 0.986 (95% Confidence interval (CI): 0.983–0.989) and 0.991 (95% CI 0.989–0.993) respectively. For interobserver agreement this was 0.982 (95% CI 0.979–0.985) (AIO) and 0.979 (95% CI 0.957–0.982) (conventional). For both the AIO and conventional windows, intra- and interobserver agreement were dependent on size, sharpness and reader experience. Measurement variability decreased with increasing lesion size. Regarding sharpness, inter- and intra-observer agreement ranged from 0.986–0.989 (AIO) and 0.985–0.992 (conventional) for well-defined lesions and from 0.978–0.983 (AIO) and 0.974–0.991 (conventional) for ill-defined lesions.

**Conclusions:**

Lesion diameters were consistently smaller on the AIO-window compared to conventional window settings. Overall intra- and interobserver variability rates were similar for the AIO-window and conventional window settings. We conclude that the AIO-window offers a reliable and reproducible alternative for measurement of thoracic lesions.

## Introduction

The (r) evolution of multidetector Computed Tomography (CT) has caused a dramatic increase in the number of images to be read by radiologists. The transition from a limited number of printed films to large volumetric datasets on Picture Archiving and Communication Systems (PACS) with numerous image processing tools and the expanding use of diagnostic medical imaging have contributed to this increase. Moreover, imaging studies are generally assessed in different window settings, allowing optimal evaluation of specific structures. Thoracic oncology studies are assessed in mediastinal or soft tissue window, lung window, and bone window settings with preset window width (W) and level (L) values in Hounsfield units. Typical settings are: W:1500 L:-600 for lung, W:350 L:50 for mediastinum and W:1800 L:400 for bone. Depending on individual preferences and available equipment, these numbers may show some variation. In the past, different image processing techniques have been developed to simultaneously display the full range of grayscale values without any need to change window width or level settings. A combined window theoretically has the potential to accelerate the image review process, since there would be fewer images to read and evaluate. Furthermore, by combining different window settings, the relationship between abnormalities and surrounding structures can be more clear, for example the relationship between a pleural mass and the rib cage, mediastinal or vascular extension from a pulmonary mass, ... Mandell and colleagues described the possibilities for a combined window in the setting of emergency radiology, oncology and nuclear medicine [1]. In chest imaging, these authors demonstrated the possible role for evaluation of diseases that contiguously affect multiple compartments, including aggressive infections, metastatic cancer and penetrating trauma [2].

This idea is not new. Postprocessing techniques such as histogram equalization [3], nonlinear CT windows [4], adaptive histogram equalization [5] companding [6] and blending [1, 7], have been investigated in the past. In contradistinction, the AIO-window is a newly developed image processing technique, derived from a commercial multiscale enhancement software for digital X-rays (MUSICA™, Agfa Medical Imaging). It fuses conventional CT window settings into a single ‘All-In-One’ (AIO) window. This single AIO-window allows visualizing the full range of CT densities without the need to adjust window width or level. It was designed for comparison and follow-up of CT studies in oncology. It was not intended for tumor staging, nor for dedicated assessment of solitary pulmonary nodules. In the imaging evaluation process of (thoracic) oncology studies, lesion detection, lesion measurement and lesion characterization are crucial elements. Previous research has shown that lesion detection on a single AIO-window is at least as good as on multiple conventional window settings [8]. CT measurements are essential in tumor imaging and determining response to therapy. Therefore the goal of this study is to investigate if lesion measurement on the AIO-window is as reliable and reproducible as on conventional window settings.

## Materials and methods

### Case selection

Approval for this study was obtained from the Institutional Review Board and informed consent requirement was waived. For this retrospective study, we selected 60 consecutive contrast-enhanced chest CT studies (GE Lightspeed VCT 64-Slice, Milwaukee, Wisconsin, USA) with 3 mm reconstructions, in standard and lung kernel, from a thoracic oncology population. We selected cases from the institutional PACS, starting from January 1st, 2016 to exclude bias. Furthermore, studies that were initially reported by the readers were excluded. We included 34 men and 26 women, with mean age of 64 years (range 35 to 82 years). Cases cover a broad spectrum of imaging findings, as one might experience in routine practice in a thoracic oncology patient population mainly including primary lung cancer.

### Image processing: ‘all-in-one’ window

**Fig. 1:**
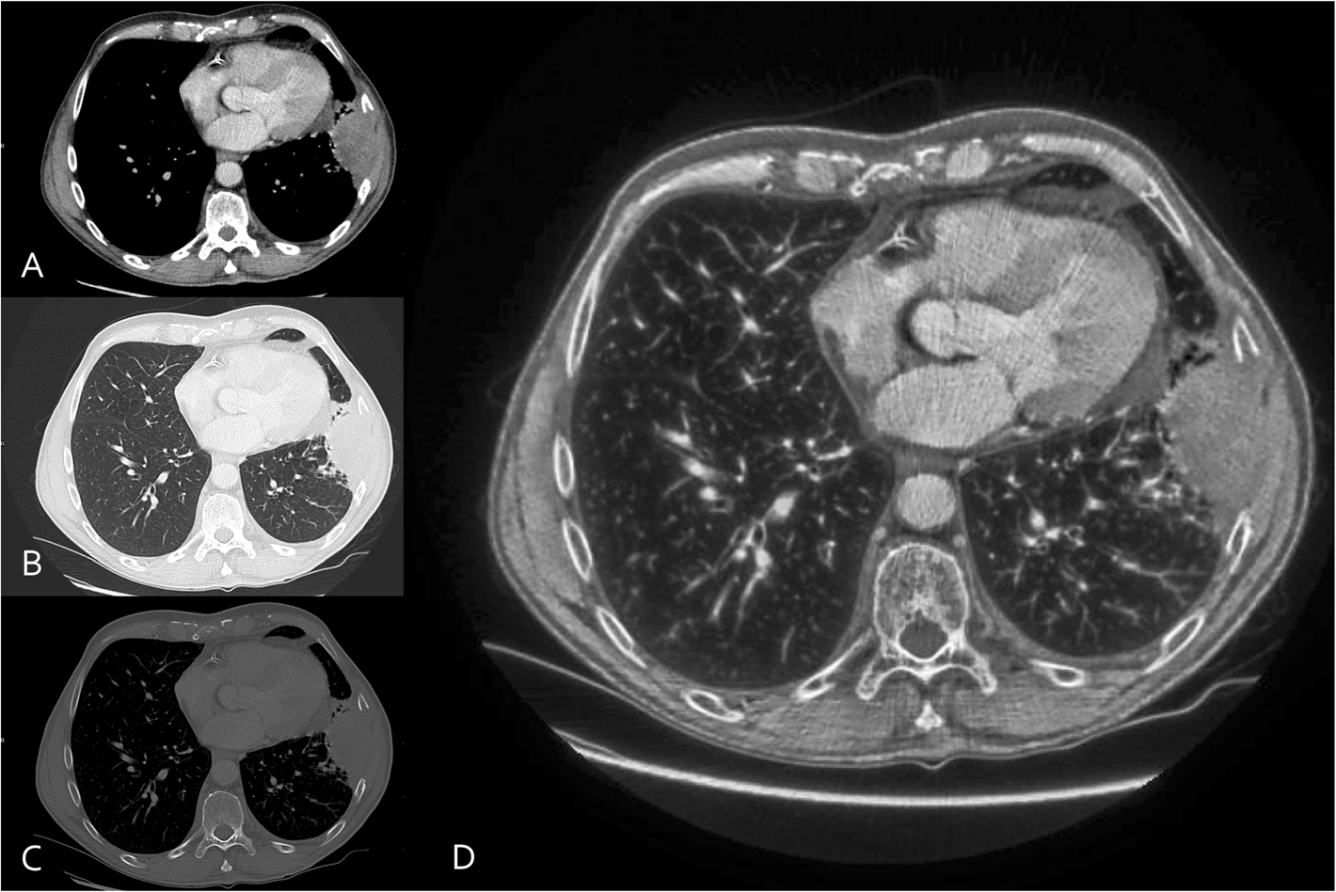
Axial contrast-enhanced CT-images (3 mm CT-reconstructions) in a 70-year-old man with stage IV lung adenocarcinoma with large mass at the left lower lobe with chest wall involvement and bone erosion. Images at the same level are shown in mediastinal (**a**), lung (**b**), and bone (**c**) window setting as well as a single AIO-window (**d**)

**Fig. 2:**
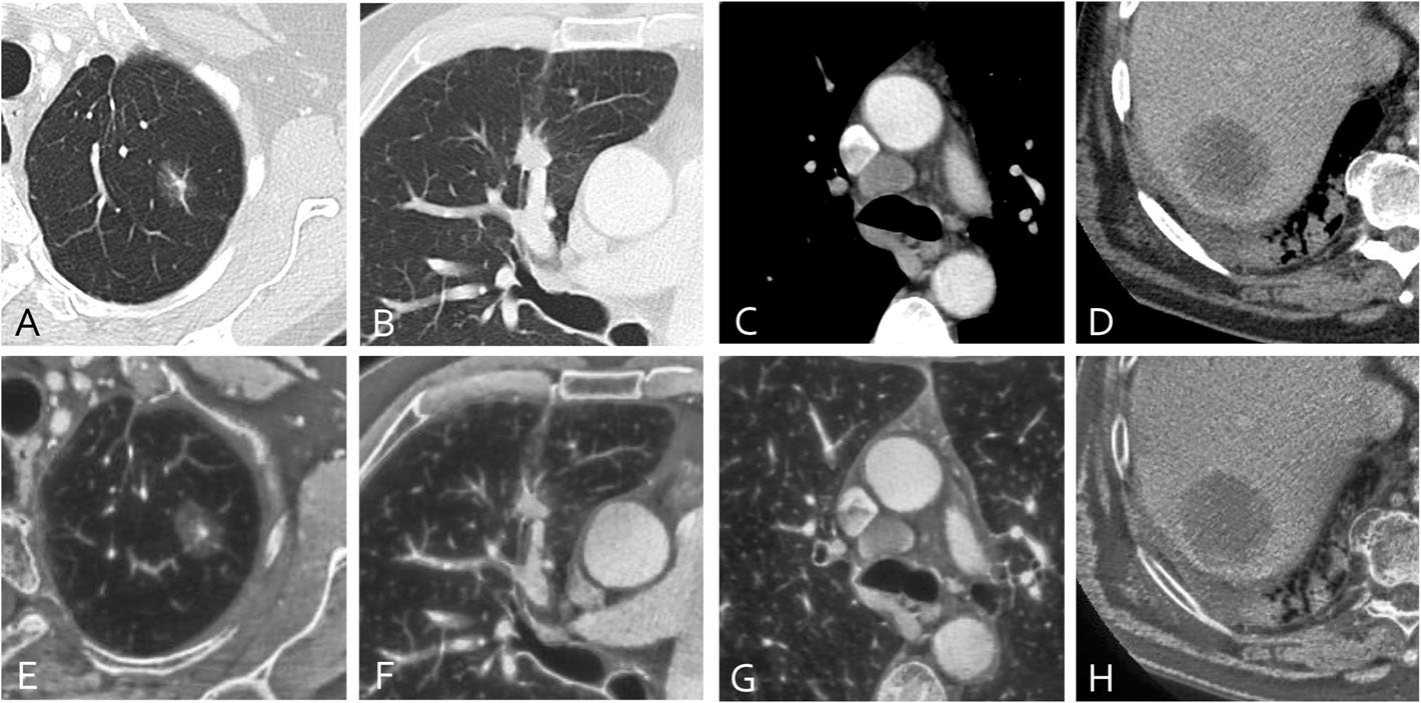
Four lesions in 4 different patients are displayed in conventional window setting and corresponding AIO-window: axial images of a part-solid nodule (**a**, **e**), spiculated solid nodule (**b**, **f**), mediastinal adenopathy (**c**, **g**) and liver metastasis (**d**, **h**) are shown in lung and soft window (upper row) with corresponding image on the AIO-window (lower row)

After anonymization of the 60 selected CT-studies, axial 3 mm DICOM-series were transferred to apply the AIO-window setting. In this novel window, multiple conventional CT windows are fused into a single AIO-window, using an image processing technique derived from a commercial multiscale enhancement software for digital X-rays (MUSICA™, Agfa Medical Imaging) (Figs. 1 and 2). The AIO-processing transforms the slices from a single reconstructed soft tissue series into a multiscale data structure that represents image contrast-detail at several scales of resolution. Subsequently, specific contrast-detail enhancement is applied to all the layers of the multiscale representation of the slices. The amount of enhancement is controlled as a function of original multiscale pixel value, Hounsfield value and scale in order to obtain a resulting contrast-detail and brightness that is most suited for the examination type, in this case oncologic chest/abdomen studies. The resulting enhanced multiscale data structure is finally converted into a readable series of grayscale slices. Typical challenges associated with CT images such as the huge dynamic range of the Hounsfield scale and the presence of strong pixel value transitions across different tissue types, have been addressed by a patented decomposition technique called Fractional Multiscale Processing®. This technique makes it possible to render all tissue types with a contrast-detail that is comparable to the original contrast-detail, whereas this is not possible with conventional blending techniques such as mentioned in [1, 7]. In blending techniques as described by Mandell et al. the soft tissue image is used as a substrate which is modified in the Hounsfield subregions of the lungs and the bone by either pixelwise addition or replacement, respectively using Photoshop. This method is able to provide images appearing similar to conventional windowing in those regions where the windows do not overlap. In transition zones however, artefactual lines may be seen, such as the interface between lungs and mediastinum. In contrast to blending techniques, the AIO image is obtained by applying specific modifications to the transformed image, instead of modifying the pixel values of the original image. The modifications applied to the transformed image are such that the pixel value range of the AIO image fits within the display range with appropriate detail contrast for all image regions, whether lung parenchyma, soft tissue or bone. The resulting grey scale of the AIO image is not identical to the Hounsfield range, but detail contrast and grey scale order is respected to the extent that the result appears natural, without creating artefactual contours in the overlap zones.

### Lesion selection

A senior thoracic radiologist with (AS) selected lesions in both chest and upper abdomen on CT slices with conventional window settings. All measurable lesions were highlighted, with exclusion of the following: coalescing lymph nodes, lesions forming a conglomerate mass, masses confluent with mediastinal structures. When numerous lesions were present in a single organ, a maximum of 10 lesions was marked to minimize the risk of confusion. This resulted in a final list of 368 measurable lesions. The number of lesions varied per examination, ranging from 1 to 21, with lesions located both in chest (*n* = 281) and upper abdomen (*n* = 87). A detailed overview of lesion characteristics is presented in Table 1. We did not include bone metastases in our study, since the number of lytic bone metastases with measurable soft tissue component was too limited for statistical analysis. Lesions were annotated with case and lesion number and a ‘snapshot’ of the lesion was saved on PACS. This method guaranteed accurate lesion identification and allowed easy recall during measurement. Subsequently the same lesions were highlighted and annotated on the AIO-window. For each lesion following features were listed: organ, sharpness (well-defined or ill-defined) and the window on which the lesion would be evaluated on conventional window settings.

**Table 1:** Overview of lesion characteristics

Total number of lesions	368
**Window settings on which lesions would be measured**	
- Soft tissue window	234/368 (63.6%)
- Lung window	134/368 (36.4%)
**Location**	
- Chest	281/368 (76.4%)
- Abdomen	87/368 (23.6%)
**Lesion sharpness**	
- Well defined	197/368 (53.5%)
- Ill defined	171/368 (46.5%)
**Organ**	
- Thyroid	9/368 (2.4%)
- Thoracic adenopathy	91/368 (24.7%)
- Breast	2/368 (0.5%)
- Mediastinum	7/368 (1.9%)
- Lung mass	27/368 (7.3%)
- Solid pulmonary nodules	111/368 (30.2%)
- Subsolid pulmonary nodule	8/368 (2.2%)
- Pleura	24/368 (6.5%)
- Liver	51/368 (13.9%)
- Galbladder	1/368 (0.3%)
- Pancreas	2/368 (0.5%)
- Spleen	1/368 (0.3%)
- Kidney	19/368 (5.2%)
- Adrenal glands	11/368 (3.0%)
- Abdominal adenopathy	4/368 (1.1%)

### Lesion evaluation

Six readers participated in the study: 2 senior staff members experienced in thoracic imaging (AS, MJS) with >10 year experience, two junior staff members (SN, AVH) with 2 years of experience and two senior radiology residents (KC, EL) with the same level of expertise in chest imaging. The senior staff member who selected and marked lesions, was one of the study readers. There was however a 6-week period between lesion selection and first measurement evaluation. Readers were blinded for any clinical information and diagnosis. They were instructed to measure lesions according to the current Response Evaluation Criteria in Solid Tumors (RECIST) 1.1 with rounding up to the closest millimeter [9]. For each reading session, readers had to choose the image slice on which they thought the lesion showed the largest diameter. Each reader performed the measurements 4 times, twice on the AIO-window and twice on the conventional window settings. A waiting period between subsequent readings of 8 days or longer was respected. Measurements were performed with electronic calipers on a clinical PACS workstation, with reading conditions similar to routine daily practice. To exclude bias regarding morphology and borders of lesions, lesions were first measured on the AIO window and subsequently on conventional window settings. At the time of repeat measurement, readers did not have access to previously recorded measurement data, nor to the slice of previous measurement.

### Statistical analysis

Since there is no ground truth or reference size, lesions were assigned to size categories (<10 mm, 10–20 mm, >20 mm) based on the average lesion size measured on the conventional window settings by the 6 readers at 2 distinct time points. Intra- and interobserver agreements were assessed by calculating the intraclass correlation coefficient. Bland-Altman plots were created to show the agreement between lesion size measurements on the conventional (CONV) and the AIO-window [10]. Since there is no gold standard of lesion size, the average lesion size was calculated as (AIO + CONV)/2 and plotted on the x-axis, where AIO and CONV are averaged across all 12 measurements on the AIO and conventional windows respectively. The percentage change in lesion size, calculated as (AIO-CONV)/mean was plotted on the y-axis. Statistical analysis was performed with SPSS (version 23.0; IBM, New York, NY). A one-sample t-test was conducted to assess the difference between the two measurement methods. *P* < 0.05 was considered indicative of a significant difference.

## Results

**Fig. 3:**
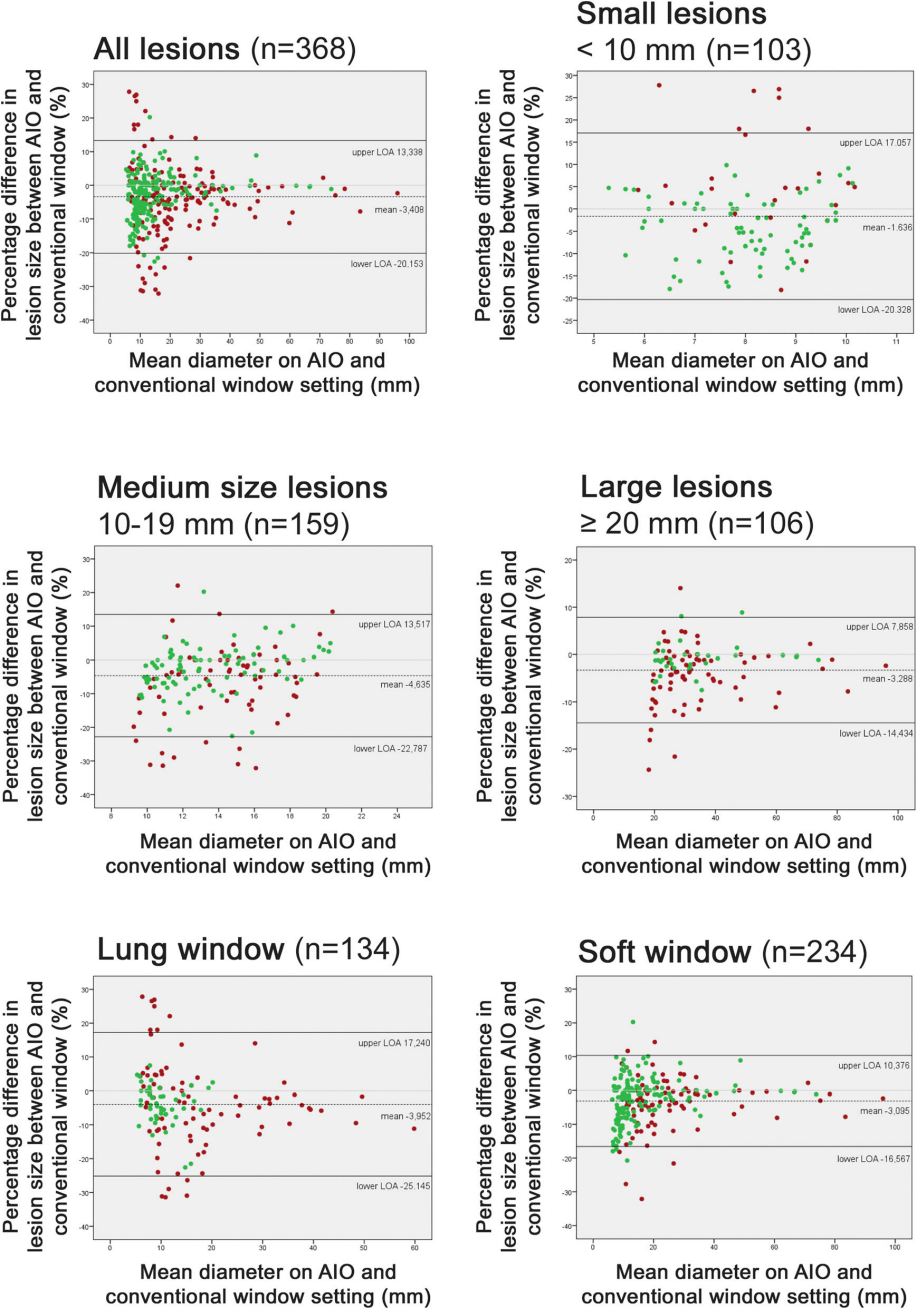
Bland-Altman plots of measurements averaged across all radiologists comparing AIO and conventional window settings (CONV). Top and bottom solid lines show the 95% limits of agreement, dashed line the mean difference. Red points are ill-defined lesions, green points are well-defined lesions

We measured 368 lesions, twice on the AIO-window, and twice on the conventional window setting, resulting in 1472 measurements per reader and 8832 measurements for all readers combined. Table 2 shows the intraclass correlation coefficients for respectively intra- and interobserver variability on both the AIO and conventional windows. Overall intra-observer agreement rates were 0.986 (95% CI:0.983–0.989) for the AIO-window and 0.991 (95% CI:0.989–0.933) for conventional window settings. Interobserver agreement rates for the AIO-window were 0.982 (95% CI:0.979–0.985) and 0.979 (95% CI:0.975–0.982) for conventional window settings. Subanalysis was performed taking into account reader experience level, window settings (lung or soft tissue window), lesion contour (well-defined or ill-defined), combination of window settings/lesion contour, and lesion size. Regarding reader experience level, intra-observer agreement on the AIO window and interobserver agreement on the conventional windows was higher for senior radiologists than for residents and junior staff members. Interobserver agreement for the AIO and intra-observer agreement for conventional window settings was higher for residents than for junior and senior staff radiologists. In general, correlation intervals overlapped, indicating a lack of significant difference. Regarding window settings, both intra- (0.987) and interobserver (0.983) agreement on the AIO-window was better for lesions that would be measured in soft tissue window settings (e.g. lymph nodes, liver lesions, …) than for lesions that would be measured in lung window setting (0.980 and 0.975 respectively) (e.g. pulmonary nodules). As expected, intra- and interobserver agreement of ill-defined lesions was lower for both AIO and conventional window settings (range, 0.978–0.991), compared to well-defined lesions (range, 0.985–0.992). For combination of lesions, intra- and interobserver agreement on both AIO and conventional window setting was highest for well-defined lesions, to be measured on soft tissue window settings (range, 0.985–0.993). Although one might expect that agreement would be lowest for ill-defined lesions in lung window settings, agreement (both intra- and inter, on both AIO and conventional windows) was higher for ill-defined lesions (range, 0.974–0.992) as compared to well-defined lesions in lung window (range, 0.940–0.945). Intra- and interobserver agreement was higher for large lesions (≥ 20 mm) both on AIO as well as on conventional window settings (range, 0.967–0.987) as compared with smaller lesions (range, 0.658–0.720 for lesions <10 mm). Figure 3 shows Bland-Altman plots comparing lesion size on the AIO-window and conventional window settings, with regard to mean diameter, different size categories and window settings. Differences within the limits of agreement (LOA) can be attributed to measurement variation, while values occurring outside these limits are suggestive of true changes in lesion size and therefore not attributable to standard deviation.

**Table 2:** Intraclass correlation coefficients to assess intra- and interobserver variability in CT size measurement

Factors	No. of lesions	Intra-observer agreement		Interobserver agreement	
		AIO window	Conventional window	AIO window	Conventional window
Overall	368	0.986 (0.983–0.989)	0.991 (0.989–0.993)	0.982 (0.979–0.985)	0.979 (0.975–0.982)
**Experience level**					
Resident	368	0.987 (0.983–0.989)	0.993 (0.991–0.994)	0.984 (0.980–0.987)	0.971 (0.965–0.976)
Junior staff member	368	0.982 (0.978–0.986)	0.991 (0.989–0.993)	0.983 (0.979–0.986)	0.980 (0.976–0.984)
Senior staff member	368	0.991 (0.988–0.992)	0.991 (0.989–0.993)	0.981 (0.977–0.985)	0.984 (0.980–0.987)
**Window setting**					
Lung	134	0.980 (0.967–0.985)	0.991 (0.987–0.993)	0.975 (0.968–0.981)	0.982 (0.977–0.986)
Soft	234	0.987 (0.984–0.990)	0.991 (0.989–0.993)	0.983 (0.980–0.986)	0.977 (0.973–0.982)
**Sharpness**					
Well-defined	197	0.989 (0.985–0.992)	0.992 (0.989–0.994)	0.986 (0.983–0.989)	0.985 (0.981–0.988)
Ill-defined	171	0.983 (0.977–0.988)	0.991 (0.987–0.993)	0.978 (0.973–0.983)	0.974 (0.967–0.979)
**Combinations**					
Lung, well-defined	49	0.940 (0.897–0.966)	0.943 (0.903–0.968)	0.945 (0.918–0.965)	0.943 (0.916–0.964)
Lung, ill-defined	85	0.979 (0.967–0.986)	0.992 (0.988–0.995)	0.974 (0.965–0.982)	0.982 (0.975–0.987)
Soft, well-defined	148	0.990 (0.985–0.993)	0.993 (0.990–0.995)	0.987 (0.983–0.990)	0.985 (0.982–0.989)
Soft, ill-defined	86	0.983 (0.974–0.989)	0.989 (0.982–0.993)	0.978 (0.970–0.984)	0.969 (0.958–0.978)
**Size (diameter in mm)**^**a**^					
< 10	103	0.720 (0.606–0.804)	0.781 (0.693–0.845)	0.649 (0.574–0.723)	0.658 (0.584–0.730)
10–19	159	0.828 (0.772–0.871)	0.881 (0.842–0.912)	0.755 (0.706–0.800)	0.729 (0.678–0.779)
≥ 20	106	0.980 (0.970–0.986)	0.987 (0.981–0.991)	0.975 (0.967–0.982)	0.967 (0.957–0.976)

Note: numbers in parentheses are the 95% CIs

^a^Lesions were assigned to size categories based on the average lesion size across all measurements in conventional window setting

When considering all 368 lesions, the LOA were − 20.2 and 13.3% with a mean difference of − 3.4% (CI − 4,3% to − 2,5%). Please note that lesion measurement is consistently smaller on the AIO-window compared to conventional window settings; this difference is statistically significant (*p* < 0.001). For small lesions (< 10 mm) mean difference is only − 1.6% (CI − 3.5 to 0.2%) but with LOA ranging from − 20.3 to 17.1%. For medium size lesions (10–19 mm) mean difference is − 4.6% (CI − 6.1% to − 3.2%) with LOA − 22.8 and 13.5%. For large size lesions ≥20 mm mean difference is − 3.3% (CI − 4.4% to − 2.2%) with LOA between − 14.4 and 7.9%. So for small lesions, the difference is not statistically significant (*p* > 0.05). For both medium and large size lesions there was a statistically significant difference (*p* < 0.001). For lesions that would be measured on lung or soft tissue window, as one might expect, the LOA for soft tissue window are more narrow (LOA − 16.6 to 10.4%) as compared to lung window (LOA − 25,2% to 17,2%).

## Discussion

Several techniques for combined CT-window settings have been described in the past, but none of these methods has ever found its way into clinical practice. First studies on this topic date from the early years of CT. Already in the 1980’s, Lehr et al. proposed a technique of histogram equalization with adjustment of image intensities to enhance contrast [3]. Some years later, Gomori et al. described a technique with non-linear window [4]. Almost 2 decades after the publication of Lehr, Fayad and colleagues studied a new method called ‘adaptive histogram equalization’, based on the initial histogram equalization technique. They investigated the usefulness of the technique in a clinical setting and were able to show a significant reduction in interpretation time for the combined window setting, but overall accuracy was insufficient for replacement of the conventional window settings by the new combined window [5]. A new technique of companding CT-images was published in 2011 by Cohen-Duwek et al., with limited clinical data [6]. More recently, Mandell et al. investigated feasibility of a ‘blended’ CT-window in patients with thoracic trauma [7]. Their results showed that the blended window approach allows for faster preliminary interpretation of axial chest CT-scans of trauma patients, with no significant difference in diagnostic performance as compared to conventional window settings. The same authors illustrated the use of window blending in aggressive infections, metastatic cancer and penetrating trauma [2].

Unfortunately, these investigations on clinical use of combined CT-window settings are limited in scope and number of patients examined.

If we want to establish the feasibility of using a combined CT-window, for example in patients with lung cancer, this would require an analysis of lesion detection, lesion measurement and lesion characterization. It is with this in mind that we decided to explore the possibilities of the AIO-window in this patient population. It has been previously shown that lesion detection on a single AIO-window is at least as good as on multiple conventional window settings [8]. However, lesion management measurement and characterization are equally important parameters in the diagnosis and follow-up of oncology patients. Therefore, in this study, we investigated the aspect of lesion measurement. Evaluation of response remains size-dependent and anatomic measurements are a crucial element in the evaluation of oncology studies [11]. Since clinical decisions are often based on CT measurements, accuracy and reproducibility of these measurements, with low rates of intra- and interobserver variability, are vital. It is known that multiple factors contribute to the variability of measurements, including operator dependant and technical aspects [12–18].

In this study, using retrospective data, we evaluated if lesion measurement on a combined AIO-window is comparable to lesion measurement on conventional window settings. Readers were asked to measure 368 lesions, twice on the AIO-window and twice on the conventional window settings. Overall intra-and interobserver agreement values were consistently high for both windows. Although still excellent, intra-observer agreement was slightly higher for the conventional window setting whereas interobserver agreement was slightly higher for the AIO-window. Subanalysis according to reader experience, showed no trend and confidence intervals were overlapping. Agreement is excellent for all categories for AIO as well as conventional window settings. Our data do not support that agreement is better for experienced radiologists. This suggests that radiologists with different level of expertise can safely perform CT measurements and that this is also the case for the novel AIO-window.

It is generally accepted that measurement differences are greater in lesions that are irregular and poorly defined or when the edge of the lesion is irregular or spiculated [16, 19]. Both intra- and interobserver agreement was lower for ill-defined lesions compared to well-defined lesions. The effect was however minimal, with still excellent agreement, on the AIO-window and conventional window settings for both categories. The Bland-Altman plots on comparing mean diameter on the AIO and conventional window settings show that lesions outside the 95% limits of agreement are mostly ill-defined lesions (red dots).

Since lesions in lung parenchym and lung window settings have a different visual aspect on the AIO-window (Figs. 1 and 2) than lesions in soft tissue window settings, this was evaluated separately. As one might intuitively expect, both intra- and interobserver agreement on the AIO-window was better for the soft tissue lesions, compared to the lesions that were evaluated in lung window setting. The agreement however was still excellent. For combined evaluation of ill- or well-defined lesions and lung or soft window on the AIO-window, agreement for ill-defined lesions in lung window seems contradictory slightly better than well-defined lesions in lung window. This is probably an artefact related to the fact that the majority of well-defined lesions in lung window are small (26/49) and medium sized (23/49) whereas the category of ill-defined lesions in lung window contains mainly medium (32/85) and large size (28/85) lesions for which overall agreement is better than for the small lesions.

In general, measurement variability increases as lesion size decreases, with the greatest variability in small tumor measurements [17]. Our data confirm this effect but show no difference for the AIO-window. For both windows, intra-and interobserver variability is lowest for lesions smaller than 10 mm (with moderate agreement) and highest for lesions of 20 mm or larger (excellent agreement). Comparing mean diameter on the AIO-window with conventional window settings show that the 95% limits of agreement are much wider for smaller lesions compared to large lesions.

Comparison of mean diameter of lesions on the AIO-window with conventional window settings, shows a consistent smaller diameter on the AIO-window. Although this difference is only 3.4%, it is statistically significant. This correlates with a diameter difference of 0.6 mm, making the clinical impact probably less important. These findings suggest that lesion measurement and comparison should be performed on the same window when comparing images and deciding on the response. A possible explanation for the overall smaller diameter on the AIO-window may lie in the fact that lesions in lung parenchyma on the conventional window settings were measured in lung window setting with a hard kernel. The AIO-window algorithm uses the soft kernel images and therefore the edges of pulmonary nodules and masses may be less sharp which may impact measurement. When one might use this window in clinical practice, it should be advised not to compare measurements on the AIO-window with previous measurement on conventional window settings.

Although it was not the primary goal of our study, our data also give insight in measurement variability on conventional window settings: overall intra- and interobserver variability is excellent. Subanalysis, taking into account lesion sharpness and size, shows excellent agreement for both well- and ill-defined lesions as well as larger lesions (≥ 20 mm). For lesions between 10 and 19 mm agreement is good, for small lesions agreement is moderate. Although still excellent, agreement is slightly higher for well-defined compared to ill-defined lesions. This is what one may instinctively expect and has been demonstrated by other studies [16, 19]. In a large study with 17 radiologists, McErlean investigated intra- and interobserver variability in CT measurements in oncology [20]. Overall variability rates as well as subanalysis showing that smooth margin and larger lesion size reduce measurement variability, are within the same range in our study. Image selection is a crucial element in lesion measurement. Lesions are generally not round but have more irregular shapes. Hopper et al. showed a wide variability between different observers in their selection of metastatic foci for measurement with significant error rate in irregular and poorly defined tumors [16]. Selection of the largest long-axis diameter can differ depending on the axial slice selected. An important limitation of the study by McErlean et al. was that readers were presented with preselected images [20]. The strength of our study is that lesions were marked at the top of the lesion and readers had to select the axial slice on which they thought was the largest diameter. Taking this into account one might expect an agreement that is less good. This was however not the case.

There are several limitations to our study. First and foremost, is the absence of an ‘imaging ground truth’ for lesion size. For obvious reasons, we were not able to compare lesion sizes with surgical pathology specimens because most patients presented with metastatic disease and were not operated. Second, our study was exclusively performed on contrast-enhanced CT’s. While this doesn’t matter for measurement of lesions in lung window settings, lesion measurement in soft tissue window settings is far more difficult. Third, we have chosen a highly specific patient population of thoracic oncology patients, because the AIO-window was specifically designed with such patients in mind. Hence, our analysis included only lesions in chest and upper abdomen. We focused in our study on 3 mm slices, since these are often used in daily practice for follow-up of thoracic oncology studies, for which the AIO-window in particular has been designed. Although many lesions in our study (Table 1) were pulmonary nodules, primarily metastatic, the AIO-window should not be used for dedicated follow-up of solitary pulmonary nodules. These lesions should be measured on thin slices (e.g. 1 mm) in lung kernel. Furthermore, in the evaluation of part-solid nodules, evaluation of the solid aspect of the lesions is crucial for staging. Currently the AIO algorithm is not designed for dedicated evaluation of pulmonary nodules. Further research in this field is necessary, before implementation of the AIO-window for evaluation of solitary pulmonary nodules, both solid and subsolid. Last but not least, a single radiologist selected lesions and addressed the category of sharpness of lesions (which is subjective), creating some bias. CT-studies and therefore lesions were not assessed in random order during the different reading sessions. Although this might create some recall bias, we believe this is minimal because of the high number of lesions (368). To minimize bias on recognition of lesion morphology (in particular for lung parenchymal lesions), measurements were first performed on the AIO-window and subsequently on conventional window settings. Some readers were familiar with the AIO-window from the previous clinical study on lesion detection [8]. There was however a one-year time interval between both studies, making visual image recall less important.

## Conclusion

In conclusion, we have shown that lesion measurement on a combined AIO CT-window is reliable and reproducible in thoracic oncology chest CT studies. For both AIO as well as for conventional window settings, overall intra- and interobserver variability of lesion measurement on chest CT studies in a thoracic oncology patient population is excellent, regardless of radiologists’ expertise. Lesions with a well-defined morphology and lesions with a larger size show less variability. Once this AIO-window approach has been validated scientifically and clinically implemented, the obvious next step will be to assess the impact on radiology reading time.

## Data Availability

The data that support the findings of this study are available from the corresponding author (AS) upon request.

## Abbreviations

AIO: ‘All-In-One’
CI: Confidence Interval
CONV: Conventional Window Setting
CT: Computed Tomography
L: Level
LOA: Limits of agreement
PACS: Picture archiving and communication systems
RECIST: Response Evaluation Criteria in Solid Tumors
W: Width
